# Refractory Severe Hypertriglyceridemia With Insulin Resistance Treated by Early Therapeutic Plasma Exchange: A Case Report and Review of the Literature

**DOI:** 10.7759/cureus.96610

**Published:** 2025-11-11

**Authors:** Ashmeet Bedi, Arya Kermanshah, Sabeer Bedi, Garine Kalaydjian

**Affiliations:** 1 Medicine, Lake Erie College of Osteopathic Medicine, Erie, USA; 2 Internal Medicine, St. John's Riverside Hospital, Yonkers, USA

**Keywords:** case report, hypertriglyceridemia, insulin resistance, pancreatitis, plasmapheresis, therapeutic plasma exchange

## Abstract

A 44-year-old woman with a history of hypertension and gestational diabetes presented with a three-week history of pleuritic chest pain. Initial laboratory results revealed triglycerides (TG) > 4,000 mg/dL, glucose 452 mg/dL, corrected calcium 5.6 mg/dL, and normal lipase. In the intensive care unit (ICU), she received aggressive intravenous (IV) fluids and weight-based IV insulin, yet TG remained > 4,000 mg/dL, consistent with insulin-refractory hypertriglyceridemia. Given the risk of free-fatty-acid-mediated organ injury, two therapeutic plasma exchange (TPE) sessions were performed within 24 hours, lowering TG to 615 mg/dL and eventually to 349 mg/dL by discharge. This case highlights that insulin resistance can hinder its effectiveness in lowering lipids and demonstrates how early therapeutic plasma exchange may be a rational intervention in severe, refractory hypertriglyceridemia to mitigate the risk of organ dysfunction. This case highlights the potential role of early therapeutic plasma exchange in insulin-resistant hypertriglyceridemia before pancreatitis and end-organ damage develop.

## Introduction

Severe hypertriglyceridemia (HTG) > 1,000 mg/dL is a metabolic emergency that can significantly increase the risk of acute pancreatitis and multiorgan failure [1,2]. Insulin resistance increases hepatic very low-density lipoprotein (VLDL)- triglycerides (TG) production and worsens hypertriglyceridemia [3]. intravenous (IV) insulin is a first‑line therapy whose effectiveness depends on functional lipoprotein lipase (LPL). Pathophysiology consists of reduced hepatic responsiveness to insulin, which leads to increased hepatic glucose production and synthesis of triglycerides into VLDL particles. Overproduction of these particles exacerbates functional LPL insufficiency, impairing TG-rich lipoproteins clearance and promoting hyperglycemia, acute pancreatitis, and cardiovascular complications. When severely high levels of TGs are no longer broken down by LPL, they are instead hydrolyzed by pancreatic lipases, which release toxic free fatty acids into the circulation [1]. Along with inflammation, hyperviscosity from excessive chylomicrons in the plasma also impairs blood flow and oxygen delivery to tissues, resulting in ischemia and multiorgan dysfunction [4]. When triglyceride concentrations exceed 2,000-3,000 mg/dL, blood itself may appear turbid or even “strawberry milkshake” pink, and plasma can separate into a creamy chylomicron layer after centrifugation, a visual hallmark of extreme lipemia with direct implications for laboratory interference. 

Primary etiologies of severe HTG with TG levels >1,000-1,500 mg/dL typically result from: (1) mutations in LPL from familial chylomicronemia syndrome (FCS) and (2) multifactorial chylomicronemia syndrome (MFCS), a more common cause of severe HTG that contains genetic and secondary forms of HTG [5]. After gallstones and excessive alcohol use, HTG is the third most common cause of acute pancreatitis [1]. The risk of acute pancreatitis is around 5% for patients with TG >1,000 mg/dL, and increases to 10-20% for patients with TG > 2,000 mg/dL [1]. Cases of pancreatitis induced by hypertriglyceridemia demonstrate a greater incidence of complications, multiorgan failure, and more severe hospital courses [6].

While IV insulin remains a first-line therapy, heparin is no longer recommended due to rebound hypertriglyceridemia and the risk of bleeding [6]. In cases refractory to insulin, particularly when end-organ damage is evident or there is a high risk of acute pancreatitis or multiorgan failure, rescue therapy with therapeutic plasma exchange (TPE) may be required. Rapid reduction with TPE physically removes TG-rich lipoproteins, such as chylomicrons and VLDL, from the plasma [7]. To address the systemic effects of hypertriglyceridemia on the pancreas, this procedure further removes inflammatory plasma mediators, thereby lowering the rate of free fatty acid-induced organ dysfunction [8]. TPE can also achieve therapeutic exchange of plasma for HTG-induced acute pancreatitis, using free fatty acids, apolipoproteins, and lipoprotein lipase supplemented by donors to aid in the metabolism of TG-rich lipoproteins [9]. 

The American Society for Apheresis (ASFA) guidelines classify the use of TPE in HTG-induced acute pancreatitis as a Category III recommendation, indicating the role of apheresis therapy is not established, use should be individualized, and is not explicit [10]. The current literature concentrates on TPE after the onset of acute pancreatitis due to severe HTG when other treatment options fail. More research is necessary for the use of early TPE in insulin-resistant cases of HTG to prevent impending pancreatitis. Large contemporary cohorts also show that once acute pancreatitis is established, early TPE does not clearly improve organ-failure-free days, underscoring that benefit may lie in prevention rather than rescue [11].

We present a case of severe hypertriglyceridemia refractory to insulin, successfully managed with early therapeutic plasma exchange. This case demonstrates that insulin resistance can hinder its effectiveness in lowering lipids and exemplifies how early therapeutic plasma exchange may be a rational intervention in severe, refractory hypertriglyceridemia to mitigate the risk of organ dysfunction. This report complies with the Health Insurance Portability and Accountability Act (HIPAA); patient authorization for publication was obtained, and informed consent was obtained and documented. Institutional Review Board (IRB) approval was not required for this case report involving three or fewer patients.

## Case presentation

Patient info

A 44-year-old female with a history of hypertension, six pregnancies, complicated with gestational diabetes and three spontaneous abortions, presented to the emergency department (ED) in August of 2025, for a three-week history of pleuritic chest pain. She arrived at the ED with TG > 4,000 mg/dL, glucose 452 mg/dL, corrected calcium of 5.6 mg/dL, and normal lipase. Her severe hypertriglyceridemia prompted admission to the intensive care unit (ICU) for aggressive isotonic fluids, weight‑based IV insulin infusion, and serial labs. She denied heavy alcohol use, and there was no documented history of gallstone disease. Her body mass index was 32.9 kg/m².

Clinical finding, timeline, and diagnostic assessment

**Figure 1:**
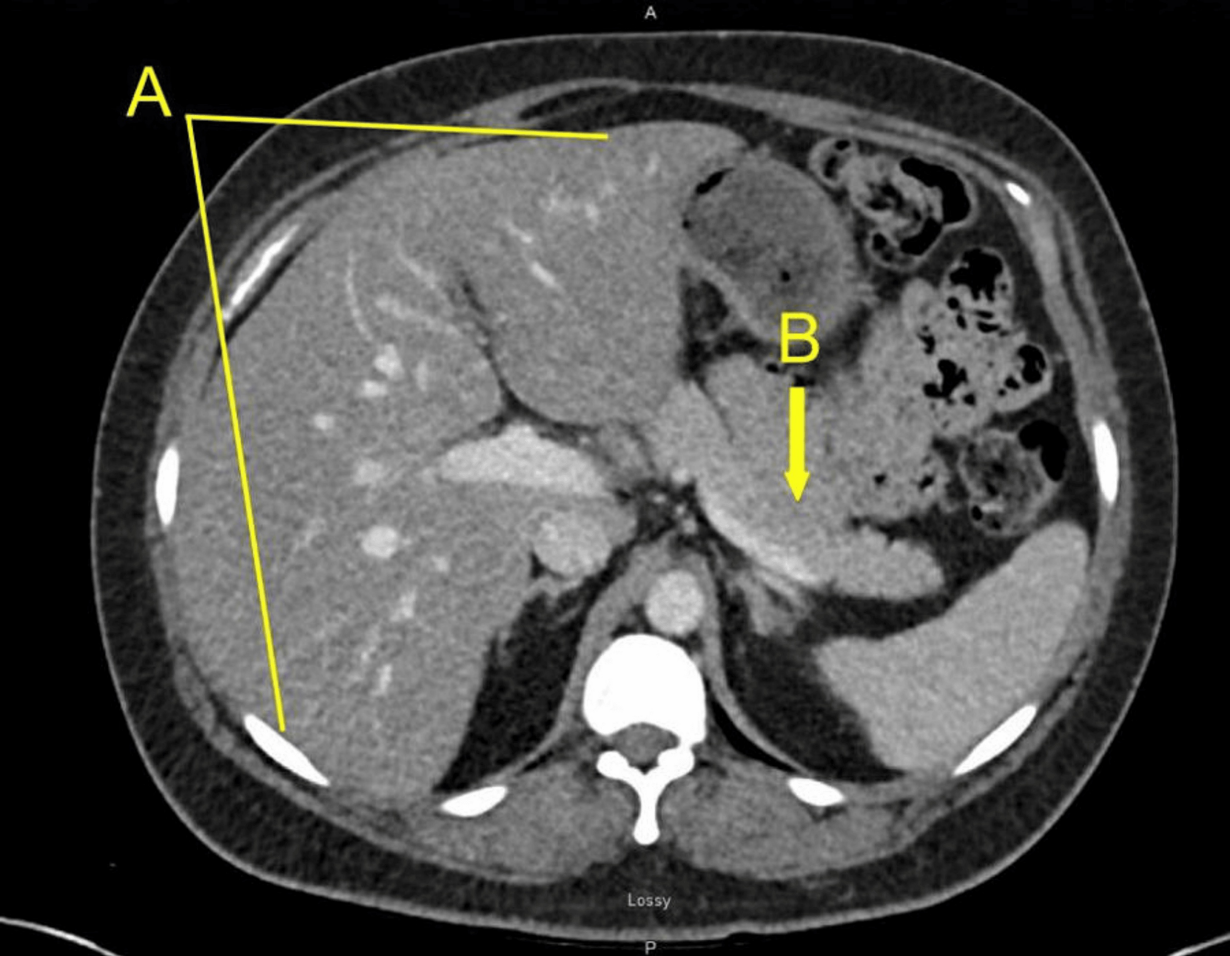
Abdominal CT Without Evidence of Acute Pancreatitis Despite Severe Hypertriglyceridemia (>4,000 mg/dL) Low-density liver (A) enlarged and hypodense compared to spleen, consistent with hepatic steatosis. No acute pancreatitis changes visible on this slice; though evaluation of pancreas (B) requires multiphase review. No focal hepatic or splenic lesion, no free fluid, no obvious acute intra-abdominal pathology in this cut.

On admission, she was afebrile, hemodynamically stable (Table 1), and her abdominal examination was benign. Initial laboratory studies revealed TG greater than 4,000 mg/dL (upper analytic limit), serum glucose 452 mg/dL, sodium 126 mmol/L, bicarbonate 22.5 mmol/L, and an anion gap of 8 mmol/L (Table 2). The corrected total calcium was 5.6 mg/dL, and creatinine was 1.1 mg/dL. Hepatic transaminases were reported as “too lipemic” to quantify reliably [alanine aminotransferase (ALT) 31 U/L, aspartate aminotransferase (AST) 57 U/L], while serum lipase was reportedly normal at 24 U/L. Hemoglobin (Hgb) was 10.7 (g/dL), Hematocrit (Hct) was 31.9 %, and white blood cell count (WBC) was 5.9 x 103/µL (Table 3). Electrocardiogram and troponin levels were non-ischemic. Chest radiograph results were pending at the time of presentation. Because severe lipemia can produce artificial hypocalcemia and spuriously normal pancreatic enzyme values, ionized calcium was obtained, and repeat chemistries were performed using dilution and ultracentrifugation techniques. A CT of the abdomen and pelvis was obtained to evaluate for pancreatitis (Figure 1) and allowed the team to monitor for signs of organ damage, which were negative. 

**Table 1:** Vitals Trend of vitals outlines the patient’s afebrile state at admission and throughout the hospital stay. Blood Pressure was mildly elevated on admission, peaked on HD2, and returned to the normal range. HD: hospital day; RR: respiratory rate.

	Normal Value	Admission/HD1	HD2	HD3	HD4	HD5
Temperature (F)	97-99	97.9	98.2	97.3	97.7	98.5
Blood Pressure (mm Hg)	<120/<80	134/88	146/89	111/75	115/68	107/63
Pulse (beats/min)	60-100	89	66	73	79	80
RR (breaths/min)	12-20	20	16	12	15	20
Oxygen, %	95-100	100	100	100	100	100

**Table 2:** Comprehensive Metabolic Panel (CMP) Baseline metabolic parameters and trend of laboratory levels over the course of hospitalization. Initial labs on admission revealed significant abnormalities, including hyponatremia (127 mmol/L), hyperglycemia (385 mg/dL), hypertriglyceridemia (>4,000 mg/dL), hypocalcemia (Ca: 5 mg/dL), and hypoalbuminemia (2.5 g/dL). With the use of therapeutic plasma exchange on HD2, progressive normalization of triglycerides, glucose, and calcium is noted for the remainder of the stay. HD: hospital day; BUN/Cr: blood urea nitrogen/creatinine; AST: aspartate aminotransferase; ALT: alanine aminotransferase; ALP: alkaline phosphatase.

	Normal Value	Admission/HD1	HD2	HD3	HD4	HD5
Sodium/ Potassium (mmol/L)	136-146/3.5-5.0	127/3.5	130/4	139/3.9	136/4.3	133/4.0
BUN/Cr (mg/dL)	7-18/0.6-1.2	11.8/0.8	8.4/0.5	<1.0/0.4	4.5/0.4	7.1/0.5
Random Glucose (mg/dL)	70-140	385	312	113	256	246
AST/ALT/ALP (U/L)	12-38/10-40/25-100	57/not reported/145	-	105/130/77	43/96/78	25/80/109
Calcium (mg/dL)	8.4-10.2	5	6.9	8.5	8.5	8.4
Albumin (g/dl)	3.5-5.5	2.5	-	4	3.6	3.8
Triglycerides (mg/dL)	<150	>4,000	>4,000	401	492	349

**Table 3:** Complete Blood Count (CBC) Trend of CBC over the course of hospitalization includes Hemoglobin (Hgb), Hematocrit (Hct) persisting below the normal range, with White Blood Cells (WBC) remaining normal throughout the stay. Platelet (Plt) count was slightly elevated at admission but remained within the normal range during hospitalization. Hgb: hemoglobin; Hct: hematocrit; WBC: white blood cells; Plt: platelet.

	Normal Value	Admission/HD1	HD2	HD3	HD4	HD5
Hgb (g/dL)	Female: 12.0-16.0	10.7	-	9.9	9.7	10.4
Hct, %	Female: 36-46	31.9	-	31.3	31.2	33.3
WBC (10^3^/µL)	4.5-11	5.9	-	8.4	6.7	6.3
Plt (10^3^/µL)	150-400	404	-	305	307	327

During the first 24 hours in the ICU, she received aggressive IV fluids, weight-based IV insulin infusion with hourly glucose monitoring, and titrated dextrose supplementation to maintain euglycemia. Per ICU protocol, potassium and phosphate were restored; while total calcium remained low, raising suspicions of pseudohypocalcemia. Despite 12-18 hours of insulin therapy and fasting, triglycerides remained above 4,000 mg/dL (decline <25%), implying poor early biochemical response or severely elevated starting TG levels. TPE was initiated on ICU days one to two. The patient tolerated well, and following the second session, triglyceride levels fell to 615 mg/dL. 

Disposition and follow‑up (hospital day five and beyond)

She was discharged on a prescription of fenofibrate (135 mg/day) and omega-3 ethyl esters (4 g/day) with a low-fat diet; TGs at discharge were 349 mg/dL; outpatient lipid/diabetes optimization was arranged. The regimen the patient was discharged with was consistent with the 2021 American College of Cardiology (ACC) Expert Consensus and the 2019 American Heart Association (AHA) Science Advisory, which recommend fibrates and prescription-strength omega-3 fatty acids in patients with severe hypertriglyceridemia to lower TG levels and reduce the risk of pancreatitis. On follow-up with the patient, she continued to be adherent to the medication regimen and had already followed up with the referred-to endocrinologist and her primary care physician for further management. She was doing well and had no symptoms or other reported findings of organ dysfunction from those visits. 

## Discussion

This case of severe hypertriglyceridemia with pseudohypocalcemia illustrates the combined effects of insulin resistance and dysfunctional LPL activity. Optimal rescue therapy when insulin fails remains TPE; however, timely implementation requires early recognition of biochemical non-response and rapid mobilization of resources. Most literature describes TPE initiated days into hospitalization after the onset of pancreatitis, whereas this case is notable for intervention before pancreatitis developed. This aligns with proposals that escalation be considered when TG decline is <25-50% in the first 12-24 h of insulin therapy or when baseline TGs are extreme [4]. Because fasting glucose and insulin were not available before insulin therapy, Homeostasis Model Assessment of Insulin Resistance (HOMA-IR) was not calculated. Given that HOMA-IR requires a fasting, insulin-naïve state and is unreliable during insulin infusions or acute metabolic stress, we used early biochemical non-response (TG decline <25-50% at 12-24h) plus persistently extreme TG levels as operational markers of insulin resistance to trigger early TPE [4].

Primary contributors to severely elevated TG levels include genetic causes such as familial hypertriglyceridemia. Familial hypertriglyceridemia Type V often exhibits TG levels in the 1,000-5,000 mg/dL range due to increased VLDL and chylomicron production, while Type I (familial chylomicronemia from LPL deficiency) typically presents with TG >2,000 mg/dL and may exceed 10,000 mg/dL [5,12]. In clinical practice, however, most cases of severe chylomicronemia are multifactorial (MFCS), in which secondary drivers such as diabetes, obesity, pregnancy, or alcohol use amplify underlying genetic susceptibility [5,12]. Her history of miscarriages raised the possibility of genetic dyslipidemia, though causality cannot be established. Dyslipidemias, including elevated TC, LDL-C, TG, and low high-density lipoprotein cholesterol (HDL-C), have been associated with miscarriage and adverse pregnancy outcomes [13], while familial hypercholesterolemia is not directly linked to miscarriage but may increase the risk of pregnancy-related hypertensive disorders and maternal cardiovascular disease. Authoritative guidelines from the National Lipid Association emphasize careful lipid monitoring and prevention of severe hypertriglyceridemia during pregnancy [14], while ACC/AHA guidelines caution that miscarriage risk is linked to statin exposure rather than the underlying lipid disorder [15]. We recommend that patients with HTG also undergo an outpatient genetic evaluation to investigate inherited dyslipidemia syndromes.

The effects of extreme HTG in this case may have additionally caused pseudohypocalcemia to occur. Severe lipemia can interfere with colorimetric assays, producing misleadingly low total calcium values, while ionized calcium remains accurate. For reliable assessment of calcium status in this setting, ionized measurements are preferred.

The toxic effects of pancreatic lipase metabolism of excess TG release toxic free fatty acids into the circulation, which would have contributed to severe acute pancreatitis, impending cardiovascular disease (CVD), and other organ failure. Early TPE yields a rapid and durable reduction in triglycerides, even in the absence of frank pancreatitis, and may have reduced the risk of pancreatitis, though causality cannot be confirmed from a single case. Because the current literature concentrates on TPE after the onset of acute pancreatitis due to severe HTG when other treatment options fail, more research is necessary to support the use of early TPE in insulin-resistant cases of HTG to prevent impending pancreatitis and systemic organ failure caused by ischemia. The case described is unique in the literature, and the management with early TPE in the absence of acute pancreatitis remains notable. Literature describing TPE often involves initiation after pancreatitis or organ dysfunction has already occurred [8,9]. This approach is often delayed by several days of unsuccessful medical therapy using IV insulin or heparin. In contrast, our patient underwent TPE within 24 h of admission before clinical pancreatitis developed, consistent with the individualized Category III stance of ASFA [10], as shown in Table 4, and the neutral findings of the large cohort in established hypertriglyceridemia-associated acute pancreatitis (HTG-AP) [11]. Based on these clinical guidelines, we would have managed her conservatively and waited. However, when noticing her TG levels were not declining, rather than waiting for organ damage to occur, we elected to initiate TPE. With the use of early intervention, we were successfully able to achieve dramatic downtrending TG levels, potentially evading end-organ injury. Furthermore, the case highlights the importance of promptly mobilizing apheresis resources and adjunct steps, including catheterization and consents, especially in resource-limited settings and off-hours. Implementation requires rapid coordination with trained personnel and the blood bank to secure apheresis equipment and calcium-containing replacement fluid (albumin or plasma).

**Table 4:** Selected American Society for Apheresis (ASFA) Category Assignments for Therapeutic Plasma Exchange (TPE) Category I disorders (e.g., thrombotic thrombocytopenic purpura, Guillain–Barré syndrome) represent first-line indications where TPE is standard and should be initiated promptly. By contrast, hypertriglyceridemia-induced acute pancreatitis is classified as Category III (Grade 1C), reflecting an unestablished role with individualized decision-making [10].

Disease (Indication)	ASFA Category / Grade	Clinical Guidance
Thrombotic thrombocytopenic purpura (TPE)	I, 1A	Emergent – start at suspicion
Guillain–Barré syndrome (TPE)	I, 1A	Early – within 7 days of onset
Hypertriglyceridemia-induced pancreatitis (TPE/LA)	III, 1C	Consider only in severe/refractory cases

By day five, triglycerides had declined to 349 mg/dL, and she was discharged on fenofibrate, omega-3 ethyl esters, and a low-fat diet with close outpatient follow-up. The patient was later contacted but declined to undergo genetic testing. Her triglyceride-lowering medications are continued to be managed by her primary care physician. Post-discharge therapy was aligned with high-authority guidance: the Endocrine Society guideline and the AHA Science Advisory support fibrates and prescription omega-3 [eicosapentaenoic Acid (EPA)/ docosahexaenoic acid (DHA) or icosapent ethyl (IPE)] for severe HTG to reduce pancreatitis risk; mechanistically, omega-3 fatty acids lower TGs primarily by reducing hepatic VLDL-TG production [2,16,17]. The use of lipid-lowering therapy such as omega-3 ethyl esters has been known to reduce the formation of TG-rich chylomicrons and endogenous VLDL, which can reduce triglyceride levels by 25-50% after a month of treatment [17]. Fenofibrates can then be further used to manage elevated TG and cholesterol levels to reduce the risk of pancreatitis [2]. Fibrates are considered for patients with elevated lipid levels due to their ability to activate peroxisome proliferator-activated receptor-alpha (PPAR-α), a promoter of LPL activity to clear TGs. Additional contemporary reviews emphasize the systemic (pancreatic and cardiometabolic) complications of severe HTG and summarize emerging agents such as apoC-III and Angiopoietin-like 3 (ANGPTL3) inhibitors [18,19]. These therapies not only reduce the production of TG-rich lipoproteins but also enhance their catabolism, presenting a viable strategy to lower triglyceride levels. 

## Conclusions

This case describes severe insulin-refractory hypertriglyceridemia with triglycerides consistently >4,000 mg/dL in a patient who presented with pleuritic chest pain but was otherwise hemodynamically stable, who had no clinical or radiographic pancreatitis. Despite aggressive IV fluids and appropriately dosed insulin infusion, triglyceride levels failed to meaningfully decline in the first 12-18 hours. Because continued lipemia at this range risks free-fatty-acid-mediated organ injury, the ICU team initiated therapeutic plasma exchange (TPE) within 24 hours. After two sessions, triglycerides fell to 615 mg/dL and ultimately to 349 mg/dL by discharge, and the patient never developed pancreatitis or other organ dysfunction.

The key takeaways are: (1) insulin resistance can blunt the expected early triglyceride response to insulin in severe hypertriglyceridemia, and (2) in that setting, early coordination for TPE, before pancreatitis or organ failure is present, may rapidly reduce triglycerides and avert progression. This case suggests that when triglycerides remain critically elevated despite initial insulin, escalation to TPE may be appropriate even before guideline-based triggers are formally met.
